# Supplementary figures and images for: Effect of custom-made and prefabricated foot orthoses on kinematic parameters during an intense prolonged run
Source: PLoS One. 2020 Mar 26;15(3):e0230877. doi: 10.1371/journal.pone.0230877 (PMC7098605; doi:10.1371/journal.pone.0230877)

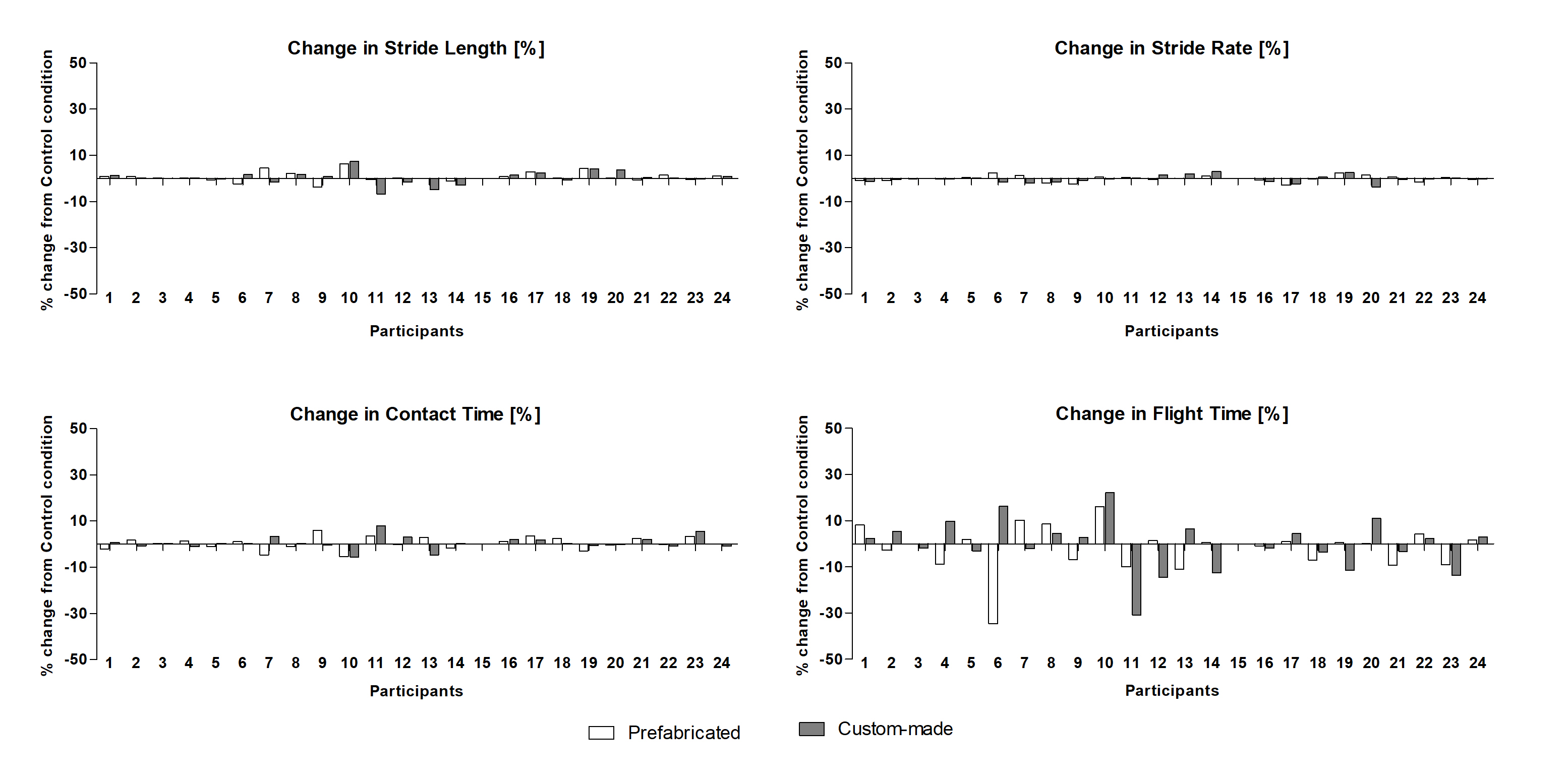

Supplement: S1 Fig — (JPG) [file pone.0230877.s001.jpg]

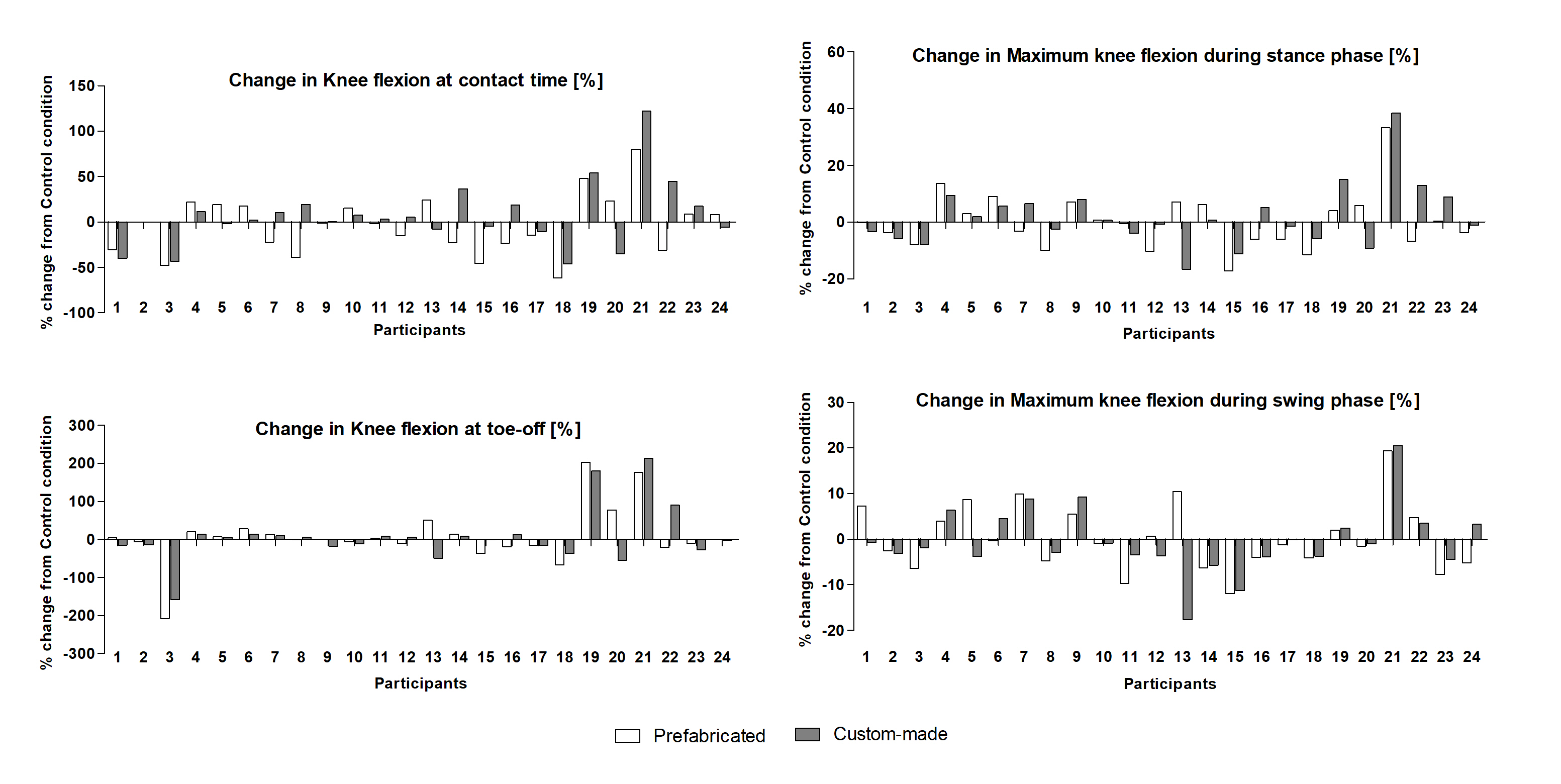

Supplement: S2 Fig — (JPG) [file pone.0230877.s002.jpg]

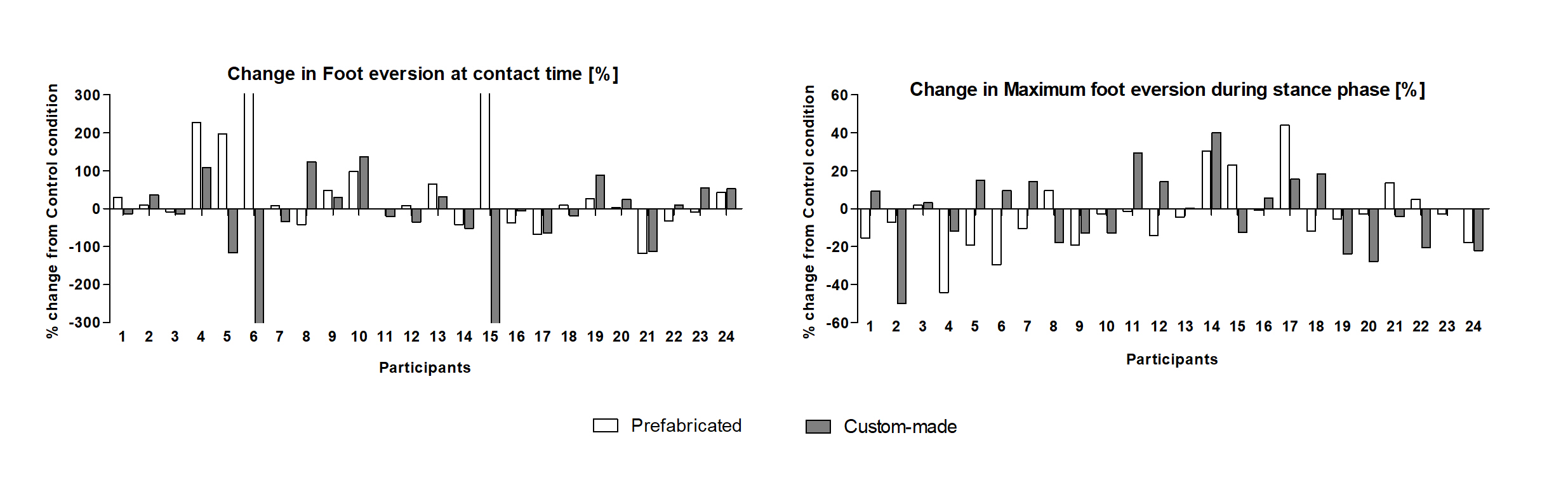

Supplement: S3 Fig — (JPG) [file pone.0230877.s003.jpg]
