# Supplementary material for: Effect of custom-made and prefabricated foot orthoses on kinematic parameters during an intense prolonged run
Source: PLoS One. 2020 Mar 26;15(3):e0230877. doi: 10.1371/journal.pone.0230877 (PMC7098605; doi:10.1371/journal.pone.0230877)
Supplement: S1 Table — (DOCX) [file pone.0230877.s004.docx]

**S1 Table. Number of participants experiencing biomechanically relevant reductions (≥10% Reduction), biomechanically relevant increases (≥10% Increase), and No Change (change between -9.9% and +9.9%) for each variable, regardless the time points of the running test, when wearing Prefabricated or Custom-made foot orthoses compared to Control condition.**

| **Variable** | **≥10% Reduction** | | **No Change** | | **≥10% Increase** | |
| --- | --- | --- | --- | --- | --- | --- |
|  | **Prefabricated** | **Custom-made** | **Prefabricated** | **Custom-made** | **Prefabricated** | **Custom-made** |
| **Stride Length** | 0 | 0 | 23 | 23 | 0 | 0 |
| **Stride Rate** | 0 | 0 | 23 | 23 | 0 | 0 |
| **Contact Time** | 0 | 0 | 23 | 23 | 0 | 0 |
| **Flight Time** | 2 | 5 | 19 | 15 | 2 | 3 |
| **Knee flexion at contact time** | 11 | 5 | 4 | 9 | 8 | 9 |
| **Maximum knee flexion during stance phase** | 3 | 2 | 19 | 19 | 2 | 3 |
| **Knee flexion at toe-off** | 6 | 10 | 10 | 7 | 8 | 7 |
| **Maximum knee flexion during swing phase** | 2 | 2 | 20 | 21 | 2 | 1 |
| **Foot eversion at contact time** | 16 | 12 | 0 | 0 | 8 | 12 |
| **Maximum foot eversion during stance phase** | 9 | 10 | 11 | 7 | 4 | 7 |
