# Supplementary material for: Effect of custom-made and prefabricated foot orthoses on kinematic parameters during an intense prolonged run
Source: PLoS One. 2020 Mar 26;15(3):e0230877. doi: 10.1371/journal.pone.0230877 (PMC7098605; doi:10.1371/journal.pone.0230877)
Supplement: Attachment — Submitted filename: Response to Reviewers.docx [file pone.0230877.s006.docx]

Thank you for submitting your manuscript to PLOS ONE. After careful consideration, we feel that it has merit but does not fully meet PLOS ONE’s publication criteria as it currently stands. Therefore, we invite you to submit a revised version of the manuscript that addresses the points raised during the review process.

In your revision, please consider revising Figs. 4-6 and/or compile them into tables for clarity, correct the grammatical errors in the manuscript, and discuss and present individual data as well as if there were responders and non-responders.

Dear editor, we are grateful for the opportunity to revise our paper. All the comments sent by the reviewers were carefully addressed. All the changes in the edited manuscript are highlighted with red text in the revised submission. We look forward to hearing from your editorial decision.

**Reviewers’ comments:**

**Reviewer #1:** The introduction and discussion are much improved now with a more narrow focus that is within the scope of the study.

Reply: We are grateful to the reviewer for this comment.

Many grammatical errors remain that should be addressed.

Reply: The manuscript has been carefully reviewed to correct all grammatical errors.

The purpose of my previous comment regarding the individual differences was to emphasize that each individual will have a very different response to a given footwear or orthotic condition. I understand the use of repeated measures statistics; however, my comment was a recommendation to show the individual data in the figures and tables (as supplements would be fine) instead of the group means and standard deviations as well as to suggest some discussion regarding if there were responders and non-responders, for example. Revealing that some subjects had more of a response than others may result in an alternative perspective or interpretation of the results than what is currently described in the discussion section.

Reply: We would like to thank the reviewer for this comment. Three figures with the percentage of change to each biomechanical variable for each participant, and one table that presents the number of participants that showed a biomechanically relevant change have been added as supplementary material. This is mentioned in the results section (Page 13, Lines 252-254):

“Percent changes of the biomechanical variables for each participant as well as the number of participants that showed a biomechanically relevant change (±10%) [36] regarding the control condition are provided in the Supplementary File.”

In addition, at the end of the discussion, a paragraph has been added regarding individual differences, as suggested (Page 18, Lines 375-385):

“From this point of view, although no differences have been found between the three foot orthosis conditions studied from a grouped perspective, in the studies of foot orthosis and footwear it is common to find a large variability of response among participants, which is masked by the overall results [36]. In the present study, as presented in the Supplementary File, the spatio-temporal parameters have not been affected by inter-subject variability, since most participants have not experienced a relevant change. However, in the angular parameters, the effects of different directions and magnitudes have been observed both among participants as well as within a single individual. In the future, it would be interesting to study if these different responses to a foot orthosis implementation could be grouped by some participant characteristics, such as the gender or the type of foot.”

**Reviewer #2:** The authors have addressed most of my previous concerns. I still feel that the data presented in Figures 4-6 would be better presented in a table form. With essentially equal means and very high deviation bars, the graphs really don't reveal anything more insightful than what could be shown in a table.

Reply: We would like to thank the reviewer for this comment. The data showed in Figures 4-6 have been presented in tables (Tables 2, 3 and 4, respectively) as suggested.
